# Effect of 3D-printed hearts used in left ventricular outflow tract obstruction: a multicenter study

**DOI:** 10.1186/s12872-022-02641-6

**Published:** 2022-04-27

**Authors:** Xianzhi Wang, Jixiang Liang, Cunfu Mu, Wenlin Zhang, Chunzhu Xue, Yang He, Gen Zhang, Dianyuan Li

**Affiliations:** 1 Department of Thoracic and Cardiac Surgery, The First People’s Hospital of Guangyuan, Guangyuan, 628000 Sichuan China; 2 The State Key Laboratory for Manufacturing Systems Engineering, Xi’an Jiaotong University, Xi’an, 710000 Shanxi China; 3 Department of Cardiovascular Surgery, The Affiliated Traditional Chinese Medicine Hospital of Southwest Medical University, Luzhou, 646000 Sichuan China; 4 Department of Cardiovascular Surgery, The Affiliated Suzhou Hospital of Nanjing Medical University, Suzhou Municipal Hospital, Gusu School, Nanjing Medical University, 26 Qian Street, Suzhou, 215000 Jiangsu China

**Keywords:** 3D printing, Left ventricular outflow tract obstruction, Cardiac surgery, Cardiac imaging

## Abstract

**Objective:**

The purpose of this research was to explore the application value of a three-dimensional (3D)-printed heart in surgery for left ventricular outflow tract (LVOT) obstruction.

**Methods:**

From August 2019 to October 2021, 46 patients with LVOT obstruction underwent surgical treatment at our institution. According to the treatment method, 22 and 24 patients were allocated to the experimental and control groups, respectively. In the experimental group, each patient’s 3D-printed heart model was used for simulated preoperative surgery, and then the Morrow operation was performed. In the control group, only the Morrow operation was performed, without simulated preoperative surgery using a 3D-printed heart model. The intraoperative and postoperative data of patients in the two groups were recorded, and the clinical data of patients were compared between the two groups.

**Results:**

The operation time, cardiopulmonary bypass time, intraoperative blood loss, hospitalization time, LVOT pressure difference (LVP), postoperative interventricular septal thickness (IST), aortic regurgitation (AR), systolic anterior motion (SAM), and postoperative left ventricular flow velocity (LVFV) were significantly lower in the experimental group than in the control group (*P* < 0.05). The inner diameter of the left ventricular outflow tract (IDLV) was larger in the experimental group than in the control group (*P* < 0.05). There was no significant difference in the postoperative ejection fraction, atrioventricular block rate or complication rate between the two groups (*P* > 0.05).

**Conclusion:**

A 3D-printed heart model for simulated surgery in vitro is conducive to formulating a more reasonable surgical plan and reducing the trauma and duration of surgery, thereby promoting the recovery and maintenance of the heart.

## Introduction

According to the hemodynamic characteristics of the left ventricular outflow tract (LVOT), hypertrophic cardiomyopathy (HCM) can be divided into hypertrophic obstructive cardiomyopathy (HOCM) and hypertrophic nonobstructive cardiomyopathy [1]. Nearly 50% of HCM patients have different degrees of LVOT obstruction due to the site and degree of myocardial hypertrophy [2, 3]. The Morrow operation is an important method for the treatment of HCM. However, due to the complex anatomical relationships in HCM, the operation is relatively difficult. The Morrow operation can only be observed through the aortic valve, and the degree of visualization is low. Therefore, excessive or insufficient myocardial tissue resection, poor obstruction relief and other serious related complications often occur. With the application of three-dimensional (3D) digital reconstruction technology in clinical disease treatment, it has become possible to obtain a reference for the formulation of surgical plans using individual patient data [4]. This study explored the application value of a 3D-printed model in the Morrow operation for LVOT obstruction.

## Materials and methods

### Case selection

From August 2019 to October 2021, 46 patients with HCM underwent surgery at Peking University International Hospital, Southwest Medical University Affiliated Hospital of Traditional Chinese Medicine and Guangyuan First People's Hospital. The inclusion criteria were as follows: LVOT pressure difference (LVP, rest or excitation) ≥ 50 mmHg; interventricular septal thickness (IST) > 18 mm; a pressure difference in asymptomatic patients at rest of more than 75–100 mmHg; severe clinical symptoms, such as exertional dyspnea, that had not improved with medical treatment; and complete data regarding the operation and follow-up. The exclusion criteria were as follows: severe organic valvular (mitral or aortic) changes found before the operation; presence of atrioventricular block before the operation; severe cardiopulmonary dysfunction; and major diseases associated with other systems. According to the treatment method, all patients with HCM previously randomized to the experimental group (22 cases) and the control group (24 cases) were analyzed prospectively for disease progression using landmark statistical analysis after the index procedure. The study was approved by the hospital ethics committee, and all patients signed informed consent forms.

### Data collection

The operation time, cardiopulmonary bypass time, intraoperative blood loss, hospitalization time, ejection fraction (EF), left ventricular flow velocity (LVFV), LVP, postoperative IST, inner diameter of the left ventricular outflow tract (IDLV), atrioventricular block rate, aortic regurgitation (AR) rate, systolic anterior motion (SAM), and complication rate were compared between the two groups.

### Treatment

In the control group, the surgical plan was made according to the conventional method, and patients in this group were treated with hypertrophic interventricular septal muscle resection and LVOT dredging through a median sternotomy. According to the preoperative imaging examination, the location of the lesion was determined, the ascending aorta and superior and inferior vena cavae were intubated, and cardiopulmonary bypass was established. After cardiac arrest, the right aortic coronary valve was pulled through the aortic root using a transverse incision approach, and the hypertrophic ventricular septum and anterior leaflet of the mitral valve were fully exposed and explored. The upper end was 5 mm below the aortic ring of the right coronary valve. The right side was 2–3 mm to the right of the midpoint of the right coronary sinus and to the left coronary sinus near the anterior mitral junction. The length of longitudinal resection was usually 50–60 mm, near the apex of the left ventricle. The abnormal chordae tendineae and papillary muscle involved in the anterior lobe of the mitral valve were removed at the same time. The abnormal connection between the body of the anterior papillary muscle and the lateral wall and interventricular septum of the left ventricle was removed to completely release the body of the anterior papillary muscle. Then, the aortotomy incision was closed, and the patient was rewarmed and weaned off cardiopulmonary bypass in the usual manner.

**Fig. 1:**
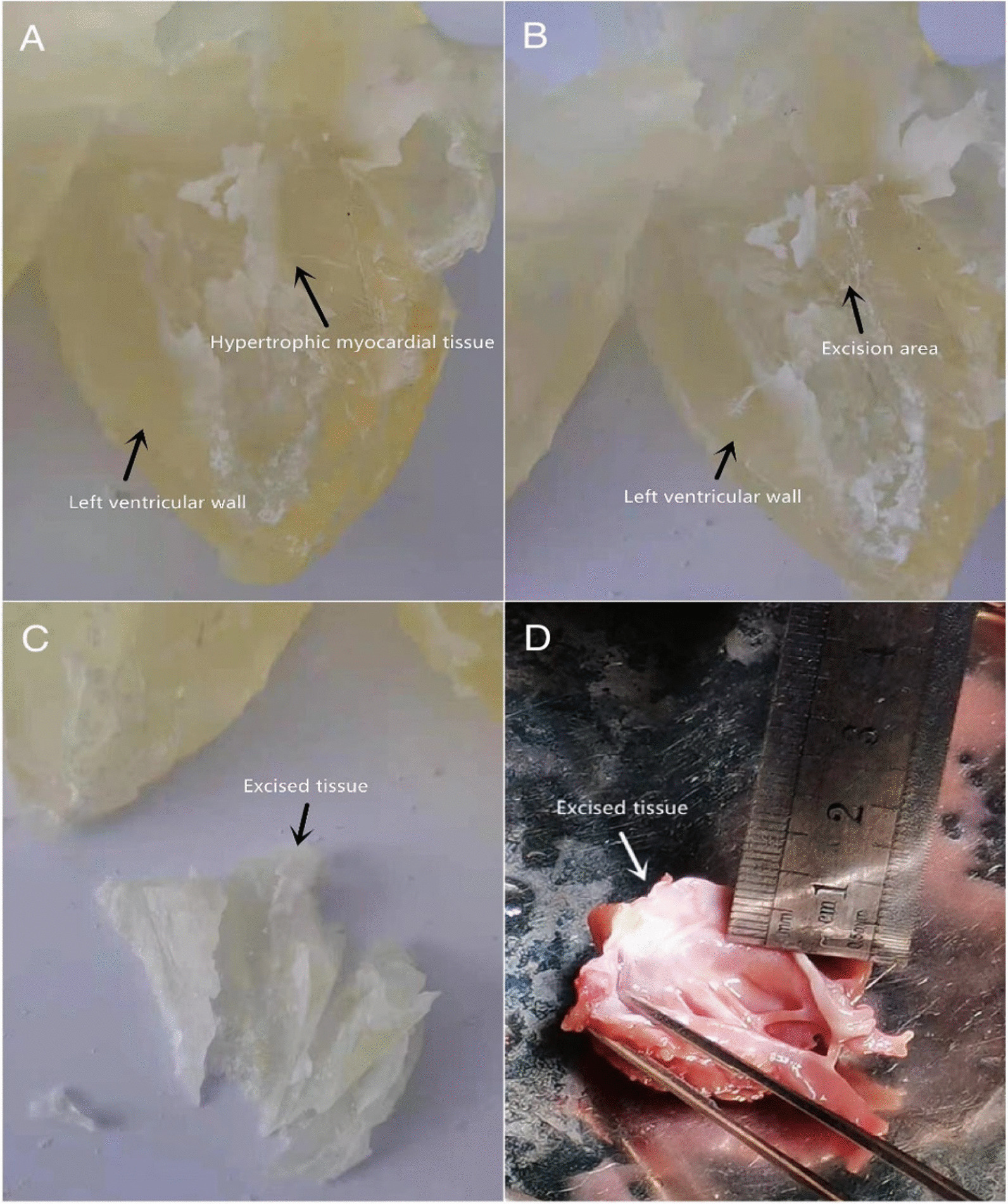
Process of hypertrophic myocardium resection facilitated using a 3D-printed model. **A** 3D-printed model of the hypertrophic myocardium before the operation. **B** 3D-printed model of the resection site after the operation. **C** Model of excised myocardial tissue. **D** Excision of myocardial tissue according to the 3D-printed model

In the experimental group, 3D printing and both a virtual and hands-on operation were performed in all cases. Computed tomography (CT) was performed before the operation, 3D reconstruction was performed with software connected to the CT system, and the data were input into a 3D printer to print a physical model of the heart affected by HOCM [5]. Cardiac CT scans were obtained using a 256-layer General Electric Revolution scanner (Revolution CT, GE Healthcare, Waukesha, WI, USA) and were recorded in Digital Imaging and Communications in Medicine (DICOM) format. The obtained DICOM raw image data were imported into the Mimics Innovation Suite 19.0 (Materialise HQ, Leuven, Belgium) for processing. To show the intracardiac structure, the blood pool area consisting of the aorta (Ao), pulmonary artery (PA), atria, ventricles, and superior and inferior vena cavae was selected using the *threshold* method as the region of interest for model generation. The myocardial model was obtained using the same method and was subtracted from the blood pool model to obtain a hollow model. It could then be cut to display the anatomical structure of the heart from multiple angles. The final blood pool volume and myocardial models were exported as stereolithography (Stl) files for 3D printing. The models were printed using an Objet Eden 260VS 3D printer (Stratasys, MN, USA) with Tango and Vero series materials. Experienced engineers made a 3D rendering of the heart through Mimics Innovation Suite 19.0, and the first assistant of the surgeon (senior doctor) verified the 3D rendering according to the CT and ultrasound data before sending the rendering to the 3D printer to print the heart model. According to the shape of LVOT obstruction and his surgical experience, the first assistant, with the help of the engineers, preliminarily performed "virtual resection" by removing the hyperplastic tissues using the 3D rendering software, recording the parameters in detail. Then, the chief surgeon performed solid resection on the 3D-printed model according to the virtual resection parameters, adjusted the range of resected tissue according to the shape of the outflow tract, and repeatedly practiced and recorded the relevant data (e.g., the site, depth, length and direction of resection). The best operation scheme was selected according to the effect of resection (Fig. 1). After the LVOT was exposed during the operation, the site of severe stenosis was found according to the 3D-printed model, and the other operations were the same as those in the control group. Mitral valve replacement was performed in patients with mitral valve leaflet organic changes or severe calcification. Transesophageal or transthoracic echocardiography was used to evaluate the SAM sign, mitral and tricuspid valve function and surgical effects. For patients with coronary heart disease, coronary artery bypass grafting (CABG) was performed with the left internal mammary artery and/or great saphenous vein after the Morrow operation.

### Follow-up

Patients were followed up with a mailed questionnaire or telephone call by contacting the referring cardiologist or general practitioner.

### Statistics

SPSS 22.0 software (SPSS, Inc., Chicago, Illinois) was used for statistical analysis. Differences in the mean ± standard deviation were determined by t tests. Count data are expressed as percentages (%).Statistical analysis was performed using the χ^2^ test, with *P* < 0.05 indicating a significant difference.

## Results

The median follow-up time was 17.23 ± 10.58 months (range: 6 to 26 months), and there were no deaths. There were no significant differences in the general data between the two groups (*P* > 0.05), which were comparable (Table 1). The operation time, cardiopulmonary bypass time, intraoperative blood loss and hospitalization time were significantly lower in the experimental group than in the control group (*P* < 0.05). There was no significant difference in the postoperative EF or atrioventricular block rate between the experimental and control groups (*P* > 0.05) (Table 2). There are three types of atrioventricular block, including that requiring pacemaker insertion. At the same time, 2 patients underwent myocardial bridge lysis, 2 patients underwent mitral valve replacement, 2 patients underwent mitral valvuloplasty, 5 patients underwent tricuspid valvuloplasty, and 1 patient underwent a modified maze procedure. There were no intraoperative deaths or deaths within 30 days after the operation in either group. CABG was performed for 1 patient with 1-vessel disease, 2 patients with 2-vessel disease and 3 patients with 3-vessel disease.

**Table 1:** Comparison of basic data between the experimental and control groups

Variable	Experimental group (n = 22)	Control group (n = 24)	t value/χ^2^-value	*P* value
Age (years, x ± s)	49.2 ± 12.2	51.6 ± 11.1	− 1.321	0.129
Male (n, %)	9 (40.9)	10 (41.6)	− 1.121	0.215
BMI	21.2 ± 2.1	20.8 ± 2.5	1.790	0.106
Preoperative EF (%)	61.0 ± 4.2	59.0 ± 4.1	1.308	0.115
LVP (mmHg)	71.5 ± 30.5	69.5 ± 42.5	2.864	0.095
IST (mm)	22.7 ± 5.3	21.9 ± 4.2	3.252	0.089
SAM (n, %)	7 (31.8)	6 (25.0)	1.581	0.125
IDLV (mm)	15.8 ± 4.7	16.5 ± 4.3	− 3.693	0.082
LVFV (m/s)	2.4 ± 0.5	2.7 ± 0.6	− 3.991	0.078

BMI, body mass index; EF, ejection fraction; LVP, left ventricular outflow tract pressure difference; IST, interventricular septal thickness; SAM, systolic anterior motion; IDLV, inner diameter of the LVOT; LVFV, left ventricular flow velocity

**Table 2:** Comparison of operative indexes between the two groups

Operative index	Experimental group (n = 22)	Control group (n = 24)	t value/χ^2^-value	*P* value
Operation time (min)	262.5 ± 59.6	281.7 ± 65.8	2.051	0.012
Cardiopulmonary bypass time (min)	79.5 ± 21.5	90.8 ± 26.2	3.894	< 0.001
Intraoperative blood loss (ml)	472.5 ± 60.6	491.6 ± 73.8	4.981	0.001
Hospitalization time (d)	7.6 ± 1.8	8.1 ± 1.6	1.894	0.023
Postoperative EF (%)	61.8 ± 8.5	59.5 ± 7.9	− 0.582	1.521
Atrioventricular block rate (%)	5.2 ± 1.8	5.5 ± 1.6	0.953	0.883

*EF* ejection fraction

### Left ventricular morphology in the two groups

There was no significant difference in the LVFV, LVP, IST, IDLV, AR rate or positive SAM sign rate between the two groups (*P* > 0.05). The measured LVFV, LVP, postoperative IST, AR rate and positive SAM sign rate were significantly lower in the experimental group than in the control group, while the IDLV was significantly larger in the experimental group than in the control group (*P* < 0.05), as shown in Table 3.

**Table 3:** Comparison of left ventricular morphological indexes

Ultrasound index	Experimental group (n = 22)	Control group (n = 24)	t value/χ^2^-value	*P* value
LVFV (m/s)	1.6 ± 0.1	2.4 ± 0.2	6.942	< 0.001
LVP (mmHg)	9.3 ± 0.3	12.3 ± 0.5	3.933	0.002
IST (mm)	8.8 ± 0.2	10.5 ± 0.3	3.912	0.001
SAM (n, %)	2 (9.09)	5 (20.83)	3.861	0.006
IDLV (mm)	30.7 ± 5.3	24.9 ± 4.2	3.257	< 0.001
AR rate (%)	6.7 ± 0.5	9.5 ± 0.5	2.134	0.013

LVFV, left ventricular flow velocity; LVP, left ventricular outflow tract pressure difference; IST, interventricular septal thickness; SAM, systolic anterior motion; IDLV, inner diameter of the left ventricular outflow tract; AR, aortic regurgitation

### Incidence of complications in the two groups

In the experimental group, there was 1 case of deep venous thrombosis. In the control group, there was 1 case of incision infection and 2 cases of deep venous thrombosis. There was no significant difference in the incidence of complications between the experimental (6.25%) and control (11.36%) groups (χ^2^ = 0.579, *P* = 0.447).

## Discussion

The Morrow operation is the gold standard for the treatment of HCM. Although Lekaditi Dimitra and others believe that medical treatment can improve the outcome, its effect is not as clear as that of the operation [6, 7]. Havndrup et al. believe that compared with other treatments, the Morrow operation is still the best in terms of postoperative effects [8]. At hospitals with experienced cardiac surgeons, the mortality rate is less than 1%. After the operation, immediate and permanent improvement in clinical symptoms can be achieved, along with a decrease in the LVP and improvement in the exercise stress response. The life span of patients treated with the Morrow operation is essentially the same as that of healthy individuals, which is better than that yielded by any other treatment method for obstruction.

### Why 3D printing?

The risk of Morrow surgery is increased due to the relatively poor visualization of the left ventricular cavity and the heterogeneity of the LVOT anatomy. The incidence of postoperative complications of the Morrow operation for inexperienced doctors is relatively high; such complications include injury to the conduction tract, damage to the atrioventricular wall, coronary artery injury, valve injury and even the occurrence of new-onset postoperative atrial fibrillation (POAF) [9].

**Fig. 2:**
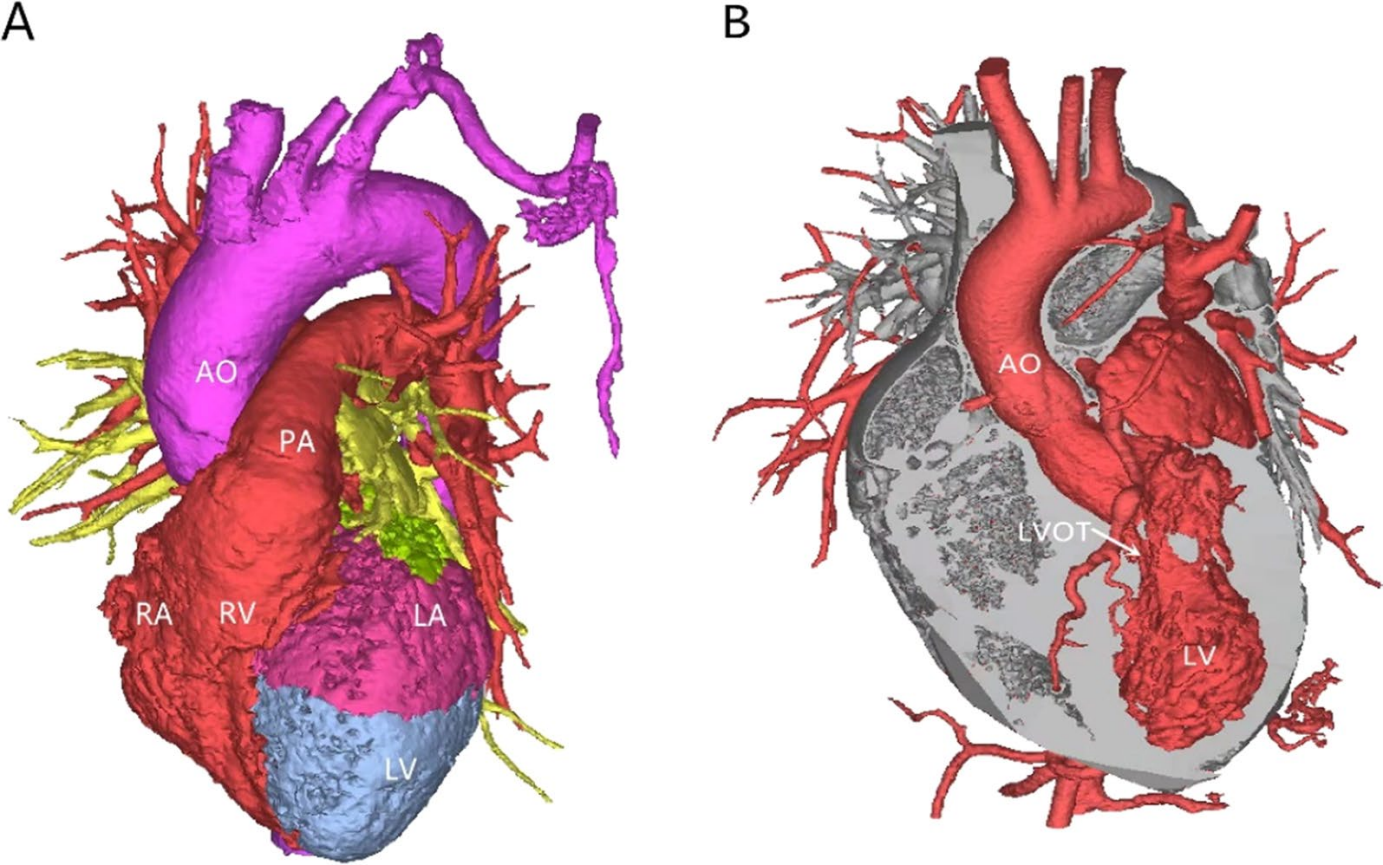
3D rendering of LVOT obstruction before surgery. **A** Use of different colors to display the heart regions directly and help distinguish tissue components. **B** Extraction of the LVOT from the 3D reconstruction of the heart to help understand the shape of the LVOT, location of stenosis, and relationships of surrounding tissues. LVOT, left ventricular outflow tract; AO, ascending aorta; PA, pulmonary artery; RV, right ventricle; RA, right atrium; LA, left atrium; LV, left ventricle

In recent years, 3D printing technology has been increasingly widely used in the surgical treatment of complex heart disease. Lee et al. believe that a 3D-printed heart model can be used to reconstruct the coronary artery anatomy and improve the understanding of coronary artery abnormalities [10]. 3D printing technology has also been widely used in the surgical treatment of congenital heart disease. In addition, satisfactory curative effects in the treatment of coronary heart disease and acquired valve disease have been achieved with the application of 3D printing technology [11]. Jivanji, SGM, et al. studied the repair of aneurysm neck occluders and right ventricular outflow tract Venus *P* valves using a 3D-printed heart model. The encouraging findings of the simulation enabled them to plan complex surgical procedures effectively and achieve successful results [12]. 3D printing can be used to visually display the geometric relationship between the hypertrophic myocardium, papillary muscle, ventricular muscle band and mitral annulus with different colors and simulate myocardial resection to better grasp the scope of hypertrophic septum resection, define the position and length of the papillary muscle and abnormal ventricular muscle band, and formulate a better surgical plan (Fig. 2).

### Intraoperative effect of 3D printing

**Fig. 3:**
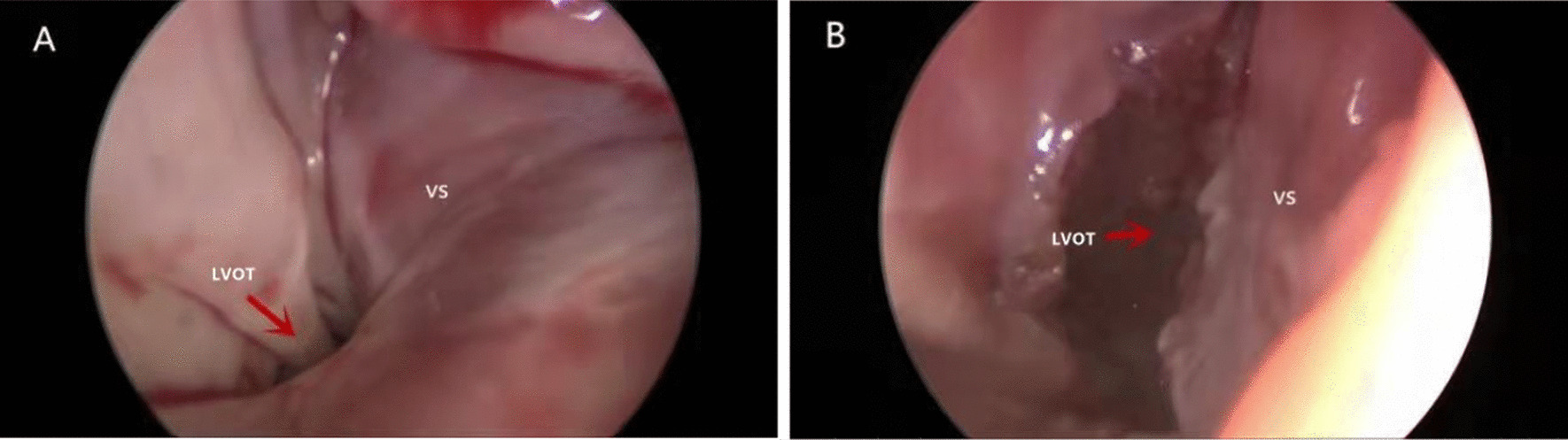
The intraoperative view before and after resection by thoracoscopy in the experimental group. **A** Thoracoscopy showing obstruction of the LVOT, indicating severe stenosis and hypertrophy of the ventricular septum. **B** Postoperative improvement in LVOT obstruction and increase in LVOT diameter by the removal of hypertrophic tissue. LVOT, left ventricular outflow tract; VS, ventricular septum

The results of this study show that the operation time, cardiopulmonary bypass time, intraoperative blood loss and hospitalization time were significantly lower in the experimental group than in the control group, suggesting that 3D printing a heart model for extracorporeal surgical simulation for patients with LVOT obstruction is helpful to shorten the operation and reduce intraoperative blood loss. The specific location, depth, and direction of resection and the best resection method for stenosis can be determined before the operation, and the Morrow operation can be simulated on this basis. Surgeons can repeatedly test the resection method on the model to determine the best resection range and depth. The results of such simulations can help to shorten the overall time needed for lesion resection during the actual operation and can be helpful for avoiding unnecessary surgical exposure and the excessive anatomical bleeding caused by the conventional operation (Fig. 3).

### Postoperative effect of 3D printing

**Fig. 4:**
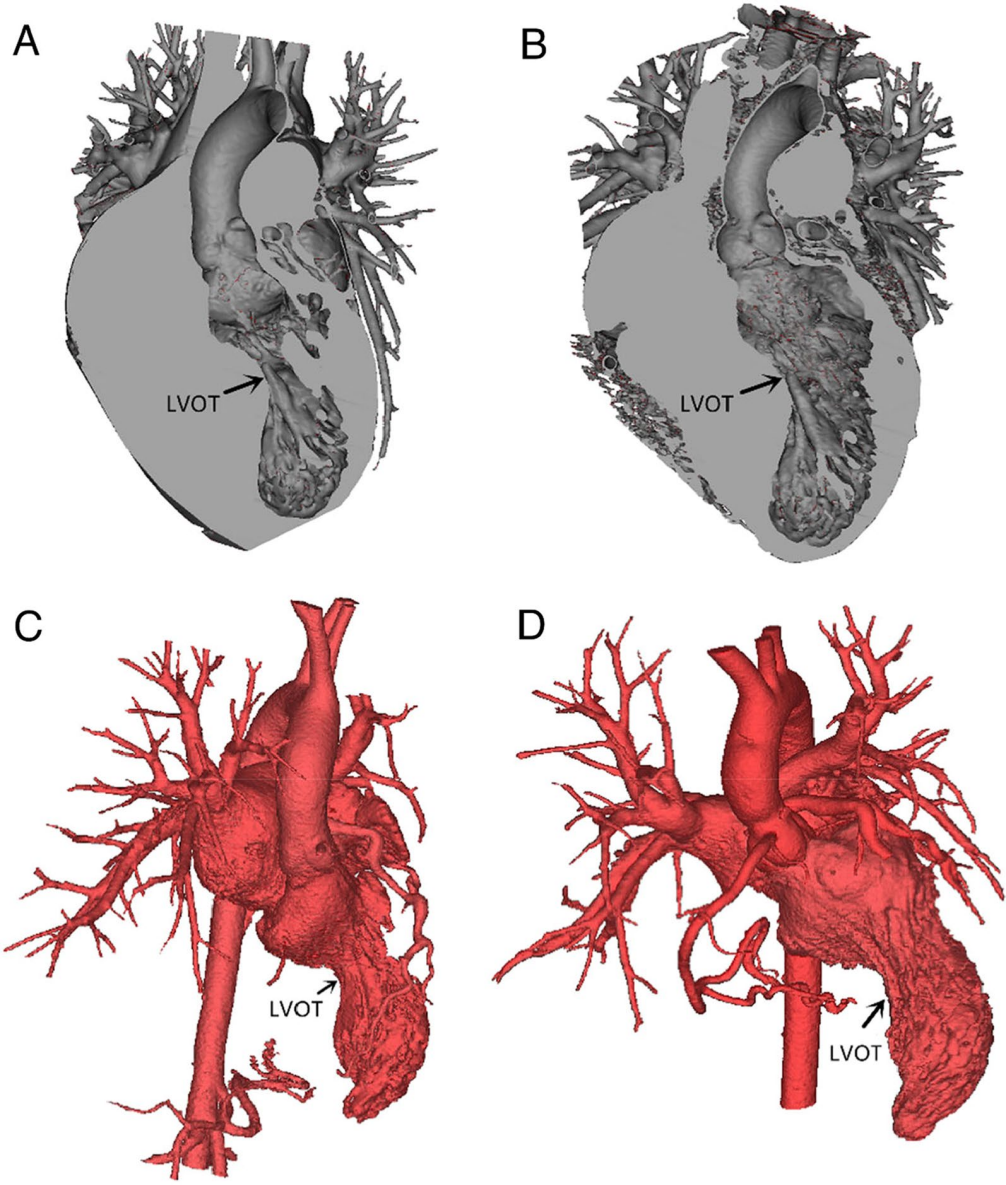
3D rendering of LVOT obstruction pre- and postoperatively. **A** Preoperative 3D rendering of coronary sections of patients with outflow tract obstruction showing severe stenosis of the outflow tract and hypertrophy of the ventricular septum. **B** Postoperative 3D rendering of coronary cross sections of patients with outflow tract obstruction showing improvement in the outflow tract, an increase in the diameter, and decreased ventricular septal hypertrophy. **C** Preoperative 3D reconstruction of the LVOT in patients with outflow tract obstruction before 3D printing indicating severe stenosis. **D** Postoperative 3D reconstruction of the LVOT in patients with outflow tract obstruction after 3D printing indicating improvement in the stenosis. LVOT, left ventricular outflow tract

The results showed that the LVFV, LVP, IST, AR rate and positive SAM sign rate were lower in the experimental group than in the control group, while the IDLV was larger in the experimental group than in the control group (*P* < 0.05). These findings suggest that 3D printing a cardiac model for simulated resection of hypertrophic myocardium in vitro in preparation for the Morrow operation is helpful for patients with outflow tract obstruction to recover a better morphology and physiological anatomy and achieve an ideal long-term effect (Fig. 4).

### Advantage of HOCM treatment in patients with coronary heart disease

It has been reported that adults with HOCM combined with CAD account for approximately 20% of HOCM cases. Huang, CH, et al. suggested that the risk of coronary heart disease with obstructive heart disease is higher whether interventional therapy or surgical treatment is applied [13]. The sudden death and total mortality rates are higher for HOCM with severe CAD than for HOCM alone. For patients with HOCM, CAD often aggravates the symptoms of angina pectoris and affects the prognosis of surgery. For patients with severe CAD, CABG should be performed at the same time. However, due to the hypertrophic myocardium, the coronary arteries are located deep in the surface of the heart or run abnormally, increasing the difficulty of locating and treating blood vessels; thus, 3D printing technology can be used for preoperative evaluation.

### Advantage of HOCM treatment in patients with valvular disease

**Fig. 5:**
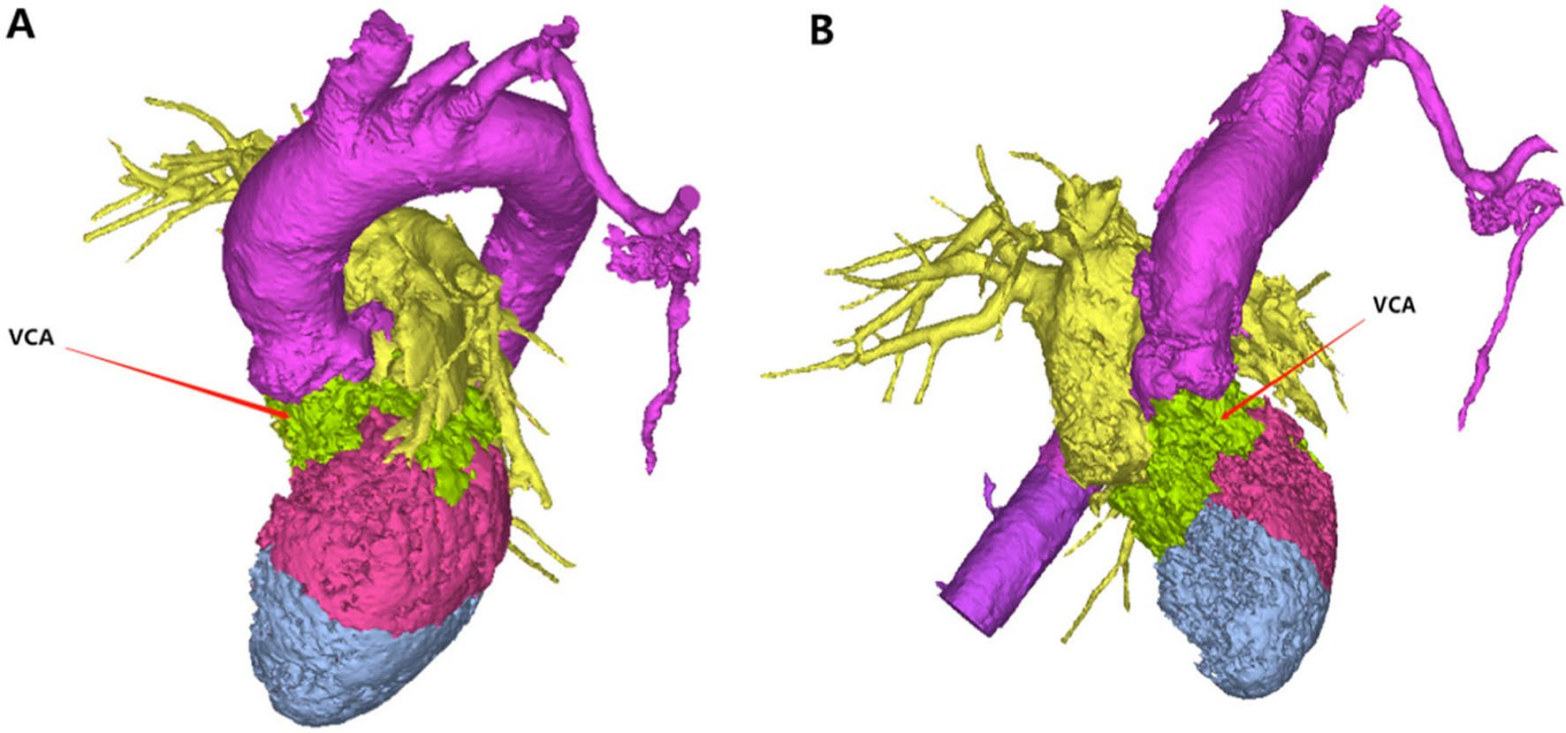
The 3D rendering of the heart showing the calcified area of the valve. **A** Coronal view showing the area covered by calcification, indicating obvious calcification of the aortic valve and mitral valve, with a wide range of involvement. **B** Sagittal view showing the relationship between the calcified valve and pulmonary artery and vein, as well as the depth of calcification invasion, which is difficult to determine intraoperatively. *VCA* valve calcification area

For patients with HOCM complicated with valvular disease, hypertrophic ventricular muscle leads to valve changes. Mitral valve problems are commonly due to LVOT obstruction. Due to SAM of the mitral valve, the valve can contact the ventricular septum and produce dynamic subaortic occlusion. This problem can be solved by the Morrow operation. Lefebvre, XP and others studied the mechanism of mitral valve SAM in HCM patients under the condition of stable blood flow, which greatly facilitates completion of the Morrow operation [14]. However, surgical treatment is required to correct diseased valves, such as those with calcification, which cannot be simply removed as can myocardial tissue [15]. In the experimental group, we performed valve replacement in two patients with obvious calcification and stenosis. Postoperatively, the valve function was normal, and the outflow tract obstruction was relieved. Cardiac ultrasound can be used to observe the degree of valve calcification and stenosis, but for patients with outflow tract obstruction, using only ultrasound to judge the change in flow velocity can easily lead to errors simply due to the problem of valve or cardiac hypertrophy. Combined with 3D printing technology, the scope and severity of lesions can be more intuitively understood. Therefore, preoperative 3D printing technology can simplify the surgical treatment of valvular disease by clarifying the scope and severity of disease and allowing the operation to be simulated (Fig. 5).

### Advantage of HOCM treatment in patients with atrial fibrillation

We previously described the case of a patient who had atrial fibrillation before surgery and needed modified maze surgery [16]. In the preoperative evaluation of patients with HOCM, it is necessary not only to evaluate the extent of resection but also to understand the shape of the nerve tracts in patients with HOCM. HOCM patients are different from general cardiac patients, with an altered nerve path due to the altered cardiac state; thus, errors such as ablation at the wrong location may affect the surgical outcome. As such, 3D printing before surgery poses certain advantages for understanding the overall shape of the heart and the patient's nerve path.

### Postoperative cardiac function

In the past, many experts have agreed that excessive cardiac tissue resection may lead to postoperative cardiac dysfunction [17]. In the present study, the LVOT diameter and wall thickness in the experimental group were significantly improved compared with those in the control group (*P* < 0.05), but there was no significant difference in cardiac function between the two groups (*P* > 0.05). However, improvement in the LVOT diameter can change the incidence of diseases related to the risk of an insufficient blood supply (e.g., stroke, myocardial insufficiency). Therefore, during the Morrow operation, with the aid of 3D printing technology, as much cardiac tissue as possible can be removed without affecting the cardiac function of patients.

### Disadvantages of 3D printing

However, because current 3D printing technology relies on data from vascular perfusion imaging, the nerve conduction bundle cannot be displayed. Lau, IWW and other researchers found that even if the 3D printing process is perfect, the inability to display the shape of the micronerve bundle remains a serious disadvantage [18]. In the attempt to remove hypertrophic myocardial tissue, it is difficult to detect the shape of the conduction beam and block conduction after the operation; this is a problem that 3D printing technology cannot solve. Therefore, in the experimental group and the control group, we found that there was no significant difference in the conduction block between the two groups (*P* > 0.05). According to our research results, compared with the conventional method, 3D printing technology can drastically reduce the LVP and the operation time in the treatment of HCM patients (*P* < 0.05). Therefore, we recommend that 3D printing of the heart be performed for all HCM patients undergoing treatment by a team with experience in 3D printing of the heart to help surgeons successfully complete the operation.

### Limitations of the study

The present study has several limitations. First, the processes of virtual modeling and surgery are complex. In this study, virtual surgery was realized with the participation of engineers and doctors; nonetheless, the process requires the support of a multidisciplinary research team. 3D printing is time consuming and expensive for practical daily use. Second, it is impossible for surgeons to completely simulate normal cardiac function by simulating surgery in vitro with a 3D-printed heart model. For example, it is not possible to evaluate the change in SAM after the operation. Therefore, at present, 3D printing technology can only be used as a reference for surgical methods and needs to be evaluated in combination with intraoperative esophageal ultrasound. However, with the development of 3D modeling technology, it will be possible to dynamically simulate cardiac ejection function after cardiac repair. Third, the sample size of this study was small, which increased the difficulty of reporting significant improvements. Although the proposed process has been proven effective in specific cases, only limited conclusions and inferences can be drawn. Fourth, this method should be applied by experienced individuals to ensure the accuracy of the model and prevent the surgeon from being given false information. Our future works will focus on automation of the 3D modeling process to remove the human aspect that is still required.

## Conclusion

3D printing a heart model can enable the surgeon to more instinctively understand the patient's heart condition and make the operation more intuitive. The use of a 3D-printed heart model for simulating surgery in vitro is conducive to creating a more reasonable surgical plan, which can reduce the trauma and duration of surgery and is conducive to the recovery and maintenance of the heart.

## Data Availability

The datasets generated and analysed during the current study are not publicly available but are available from the corresponding author on reasonable request.
